# *ABCG2* Single Nucleotide Polymorphism Affects Imatinib Pharmacokinetics in Lower Alpha-1-Acid Glycoprotein Levels in Humans

**DOI:** 10.3389/fphar.2021.658039

**Published:** 2021-04-29

**Authors:** Jin-Woo Park, Hyewon Chung, Kyoung-Ah Kim, Jong-Min Kim, In-Hwan Park, Sangjin Lee, Ji-Young Park

**Affiliations:** ^1^ Department of Clinical Pharmacology and Toxicology, Anam Hospital, Korea University College of Medicine, Seoul, Korea; ^2^ Department of Clinical Pharmacology and Toxicology, Guro Hospital, Korea University College of Medicine, Seoul, Korea

**Keywords:** imatinib (gleevec), glivec, STI-571, alpha-1 acid glycoprotein, ABCG2 (BCRP), genetic polymorphism

## Abstract

Imatinib is transported extracellularly by ABCB1 and ABCG2 efflux transporters and bound to alpha-1-acid glycoprotein (AGP) in the bloodstream. However, the clinical and pharmacokinetic effects of ABCB1 and ABCG2 on imatinib were inconsistent in the previous literature and have not been confirmed. Therefore, in the present study, we explored the effects of the *ABCG2* and *ABCB1* genetic polymorphisms on imatinib pharmacokinetics in association with plasma AGP levels in healthy subjects. Twenty-seven healthy individuals were recruited, genotyped for *ABCG2* and *ABCB1*, and given a single oral dose of 400 mg imatinib. Plasma imatinib concentrations were measured and its pharmacokinetics was assessed with respect to *ABCG2* (c.421C>A and c.34G>A) and *ABCB1* (c.1236C>T, c.2677C>T/A, and c.3435C>T) genotypes, and plasma AGP levels. AGP levels showed a strong positive correlation with imatinib pharmacokinetics. ABCG2 c.421C>A single nucleotide polymorphism showed a statistically significant effect on imatinib pharmacokinetics in low plasma AGP levels groups (<80 mg/dl); subjects with high plasma AGP levels (n = 5, ≥80 mg/dl) were excluded. The results indicate that plasma AGP levels and *ABCG2* polymorphisms modulated imatinib pharmacokinetics; however, the effects of the ABCG2 transporter was masked at high plasma AGP levels.

## Introduction

Imatinib mesylate (Gleevec, formerly STI-571) is an approved drug for chronic myeloid leukemia (CML) (Capdeville et al., 2002) and gastrointestinal stromal tumors (GISTs) that work by selectively inhibiting BCR-ABL and tyrosine kinase (Demetri et al., 2002). It has been reported that patients with poor imatinib treatment response, generally have lower systemic levels of imatinib than patients who respond well (Picard et al., 2007; Larson et al., 2008). Furthermore, plasma levels of imatinib correlate with clinical response and survival rates (Picard et al., 2007; Demetri et al., 2009). Considerable inter-individual differences have been observed in imatinib pharmacokinetics (Peng et al., 2005) and therefore variable response.

Imatinib is a biopharmaceutical classification system (BCS) Class 1 compound with high permeability and solubility (Bhattacharya, 2020). It is well absorbed with an absolute bioavailability of 98%, and it reaches C_max_ in 2–4 h (Peng et al., 2004). Imatinib is metabolized in the liver, predominantly by cytochrome P450 isoforms CYP3A4 and CYP3A5 and to a lesser extent CYP1A2, CYP2D6, CYP2C8, CYP2C9, and CYP2C19 (Gschwind et al., 2005; Nebot et al., 2010). Imatinib is also a substrate of drug efflux ATP-Binding Cassette (ABC) transporters, including ABCB1 (MDR, P-glycoprotein) and ABCG2 (BCRP, breast cancer resistance protein) (Burger et al., 2004; Oostendorp et al., 2009; Eechoute et al., 2011). These transporters are substantially involved in the absorption, distribution, and elimination of drugs through efflux.

The two drug transporters are polymorphic and their polymorphisms are known to alter substrates’ blood levels and therefore clinical effects (i.e., loss of function of the transporters) (Kim et al., 2007; Haufroid, 2011; Kim et al., 2011a; Silverton et al., 2011). Although previous investigations produced contrasting results, a review of the literature indicated that the polymorphisms of either *ABCB1* (c.1236C>T, c.2677C>T/A, and c.3435C>T) or *ABCG2* (c.421C>A and c.34G>A) possibly influenced the plasma levels and clearance of imatinib, as well as patients’ clinical outcomes (Eechoute et al., 2011). Additionally, imatinib primarily binds to plasma alpha-1-acid glycoprotein (AGP) (approximate mean free fraction: 4%), which may significantly alter drug distribution and elimination; it has been revealed that plasma imatinib pharmacokinetics is affected by AGP levels (Gambacorti-Passerini et al., 2003). Since AGP levels are known to be elevated in various physiologic (e.g., age, pregnancy, obesity) and disease conditions (e.g., inflammation, cancer) (Kim et al., 2015), the pharmacokinetics of imatinib is prone to effects of diverse underlying conditions.

Despite various studies reporting the involvement of ABCB1 and ABCG2 transporters in the disposition of imatinib (Burger and Nooter, 2004), their roles remain controversial with limited information on their effects on imatinib pharmacokinetics. In addition, to date, no study has evaluated the effects of both the AGP levels and polymorphisms of *ABCB1* and *ABCG2* transporters on imatinib pharmacokinetics. In the present study, we investigated the effects of both polymorphic *ABCG2* and *ABCB1* genotypes, and plasma AGP levels on imatinib pharmacokinetics in a controlled study of healthy subjects. We hypothesized that the effect of *ABCG2* and *ABCB1* single nucleotide polymorphism on the imatinib pharmacokinetics was dependent on AGP levels and that in low AGP levels, ABCG2 and ABCB1 transporters increased the free fraction of imatinib. We aimed at providing insight, and a clear understanding of their effects on imatinib pharmacokinetics and their relationship with the genetic contribution to interindividual variation of imatinib exposure and response.

## Material and Methods

### Subjects

Twenty-seven healthy male participants were enrolled with a mean (±S.D.) age of 24.6 ± 1.9 years (range, 23–30 years), mean (±S.D.) weight of 69.4 ± 7.6 kg (range, 54–68 kg), and mean (±S.D.) height of 174.8 ± 4.7 cm (range, 164–174 cm). All subjects were confirmed to be healthy by a physician through a detailed physical examination; 12-lead electrocardiography, serum biochemistry, hematology, and urinalysis. Exclusion criteria were: a history of, or evidence of, a hepatic, renal, gastrointestinal, or hematologic abnormality, any other acute or chronic disease, or an allergy to any drug. All subjects were nonsmokers and were not taking any medication, and provided written informed consent. The study protocol was approved by the Institutional Review Board (IRB) of Anam Hospital, Korea University, Korea (clinicaltrial.gov; NCT01270984).

### *ABCG2* and *ABCB1* Genotyping

To determine the *ABCG2* and *ABCB1* genotypes, a blood sample was obtained from each of the participants and stored at −20°C awaiting DNA extraction. Genomic DNA was isolated from peripheral leukocytes. All individuals were genotyped for the c.421C>A and c.34G>A alleles of *ABCG2* polymorphisms and for the c.1236C>T, c.2677C>T(A), and c.3435C>T alleles of *ABCB1* polymorphisms through pyrosequencing methods using a pyrosequencer (Biotage, Uppsala, Sweden), to evaluate the SNPs rapidly, precisely, and cost-effectively as described previously (Kim et al., 2010; Kim et al., 2015).

### Study Design

After overnight fasting, the subjects were given a single oral dose of 400 mg imatinib (Gleevec; Novartis Korea, Seoul, Korea) with 240 ml of water. Blood samples were collected in heparinized tubes (Vacutainer; Becton Dickinson, Franklin Lakes, NJ) immediately before drug administration (baseline) and at 0.5, 1, 1.5, 2, 2.5, 3, 3.5, 4, 5, 6, 7, 8, 10, 12, 24, 48, and 72 h after drug administration. Plasma was separated by centrifugation (1,977 g, 4°C) for 15 min, and the samples were stored at −70°C awaiting analysis.

### Determination of Imatinib Concentration in Plasma Samples

The plasma concentrations of total imatinib (both protein-bound and unbound form of imatinib) were determined using a previously validated LC-MS/MS method with slight modifications (Kim et al., 2011b). Liquid-liquid extraction (LLE) technique was used to separate the imatinib from the plasma sample. Plasma sample (0.2 ml) was added to a glass tube containing the internal standard amount of imatinib (20 μL of imatinib-d_8_ 5 μg/ml). The tube was shaken for 10 s, after which 1.5 ml methyl t-butyl ether was added to the mixture, which was then vortexed for 20 min. The organic phase was then transferred to a clean glass tube and evaporated to dryness under a flow of nitrogen gas. After reconstitution of the dry residue with 1 ml acetonitrile (50%), a 2 μl aliquot of this solution was injected onto the LC-MS/MS system, equipped with an Imtakt Unison C_8_ column (3 μm, 75 mm × 2.0 mm, Imtakt Corp., Kyoto, Japan). The mobile phase was composed of 10 mM ammonium formate: acetonitrile: formic acid (60:40:0.1, vol/vol/vol) and used at a constant flow rate of 0.2 ml/min. Quantification was performed in multiple reaction monitoring modes, with the transitions of *m/z* 494.4→217.1 for imatinib and *m/z* 502.4→225.1 for the internal standard. A linear calibration curve within the range of 5–5000 ng/ml was established for imatinib. The regression correlation coefficients of the calibration curves were >0.9999. Intra-day and inter-day coefficient of variations (CVs) was below 5%.

### Pharmacokinetic Analysis

The pharmacokinetic parameters for imatinib were determined by non-compartmental analysis using WinNonlin software (version 8.1, Certara, NJ, United States). The maximum plasma concentration (C_max_) and the time to reach C_max_ (T_max_) were estimated directly from the raw data. The total area under the plasma concentration-time curve (AUC_all_) was calculated using the linear trapezoidal rule, with extrapolation to infinity (AUC_inf_) by the division of the last measured concentration by the elimination constant (K_e_). The value of K_e_ was obtained from the slope of the linear regression of the log-linear part of the raw data. The t_1/2_ (half-life) was equal to ln_2_/K_e_ and the oral clearance (CL/F) of imatinib was estimated by dose/AUC_inf_.

### Determination of AGP Levels in Plasma

Plasma AGP concentration was measured in samples obtained immediately before imatinib treatment. AGP concentration was determined using a commercially available ELISA kit (Abcam Inc., Cambridge, MA) and the intra-assay and inter-assay CV values were 4.4% and 7.0%, respectively.

### Statistical Analysis

The data were expressed as the mean ± SD in the text and tables and, for clarity, as the mean ± SEM in figures. The statistical comparisons between the *ABCG2/ABCB1* genotype groups were made with one-way analysis of variance (ANOVA) or Kruskal-Wallis one-way ANOVA by rank, with multiple post hoc comparisons performed after the normality test. Before the ANOVA test, the genotype groups were compared by the performance of an analysis of covariance with an effective term for both *ABCG2* and *ABCB1* genotypes and with demographic data including age, body weight, and height as covariates. The possible correlation between AGP levels and imatinib pharmacokinetics parameters was assessed using parametric Pearson’s correlation coefficient. The plasma AGP levels were illustrated by a probability plot and were examined for normality of the distribution by the normality test. The data analysis was computed with the statistical program SAS 9.2 for Windows. *p* values of < 0.05 were considered to indicate statistically significant differences.

## Results

### Demographic Data

A total of twenty-seven healthy subjects were enrolled in this study, they were genotyped for *ABCG2* genotype, and *ABCB1*. The observed frequencies of *ABCG2* and *ABCB1* genetic polymorphisms in the subjects and their demographic data are presented in Table 1. The genotype groups were compared by the performance of an analysis of covariance with an effective term for both ABCG2 and ABCB1 genotypes and with demographic data including age, body weight, and height as covariates. However, the interactions between genotype and each of the covariates were not statistically significant.

**TABLE 1:** Genotype frequencies of *ABCG2* and *ABCB1* genetic polymorphisms in 27 Korean subjects and demographic data.

Gene	Genotype	N	Age (year)	Height (cm)	Weight (kg)	AGP (mg/dl)
*ABCG2*						
c.421C>A	c.421CC	12	25.4 ± 2.4	174.0 ± 5.8	69.5 ± 10.5	42.1 ± 10.8
	c.421CA	9	24.3 ± 1.2	176.0 ± 4.3	69.1 ± 3.8	53.0 ± 11.7
	c.421AA	6	23.5 ± 0.8	174.5 ± 2.2	69.8 ± 5.8	50.6 ± 7.2
	p-value		0.115	0.634	0.986	0.121
c.34G>A	c.34 GG	19	25.1 ± 2.1	175.0 ± 4.4	69.4 ± 7.6	49.4 ± 11.5
	c.34 GA	6	23.2 ± 0.4	172.7 ± 4.1	67.1 ± 4.3	44.2 ± 11.1
	c.34AA	2	24.5 ± 0.7	179.0 ± 8.5	76.5 ± 14.5	40.3 ± 12.1
	*p*-value		0.095	0.243	0.325	0.549
*ABCB1*						
c.1236C>T	c.1236CC	6	25.3 ± 3.0	175.5 ± 3.8	69.5 ± 10.4	51.1 ± 9.4
	c.1236CT	8	25.1 ± 2.0	174.8 ± 4.9	72.0 ± 7.7	38.7 ± 14.1
	c.1236 TT	13	24.0 ± 1.1	174.5 ± 5.2	67.8 ± 6.1	50.4 ± 9.0
	*p*-value		0.265	0.91	0.485	0.108
c.2677G>T(A)	c.2677 GG	3	25.0 ± 1.7	174.3 ± 1.2	72.9 ± 4.2	46.5 ± 19.1
	c.2677 GA	4	26.8 ± 3.3	176.5 ± 3.7	74.4 ± 8.4	58.6 ± 2.8
	c.2677 GT	11	24.3 ± 0.9	176.9 ± 5.0	70.7 ± 7.4	51.5 ± 7.9
	c.2677 TA	2	23.0 ± 0.0	170.5 ± 4.9	67.0 ± 7.6	30.0 ± 12.4
	c.2677AA	1	28	171	64.1	39.6
	c.2677 TT		23.7 ± 1.2	172.0 ± 4.3	65.4 ± 5.3	46.0 ± 9.8
	*p*-value		0.025*	0.194	0.107	0.098
c.3435C>T	c.3435CC	8	26.3 ± 2.6	175.0 ± 3.2	71.3 ± 9.2	50.0 ± 12.8
	c.3435CT	14	24.2 ± 1.1	175.1 ± 6.0	69.3 ± 7.6	47.6 ± 11.9
	c.3435 TT	5	23.2 ± 0.4	173.6 ± 1.9	66.8 ± 4.5	46.0 ± 9.8
	*p*-value		0.005*	0.834	0.605	0.861

*p< 0.05

### Effects of Polymorphic ABCG2 and ABCB1 Genotypes on Imatinib Pharmacokinetics

Based on our study results *ABCG2* genotypes, c.421C>A and c.34C>T polymorphism did not influence imatinib pharmacokinetics. *ABCG2* c.421C>A, exhibited a gene-dose dependent trend on imatinib pharmacokinetics marginally (CL/F, *p* = 0.049, Table 2). Similarly, *ABCB1* genotypes, c.1236C>T, c.2677G>T/A, and c.3435C>T polymorphisms did not show influence on the pharmacokinetic parameters of imatinib in this study population (Table 2).

**TABLE 2:** Comparisons of pharmacokinetic parameters of imatinib by *ABCG2 and ABCB1* genotypes.

*ABCG2* c.421C>A	c.421CC (n = 12)	c.412CA (n = 9)	c.421AA (n = 6)				*p*-value
T_max_ (h)	3.1 ± 0.7	3.1 ± 0.8	2.9 ± 0.6				0.890
C_max_ (ng/ml)	1611.3 ± 328.4	1878.1 ± 389.9	2001.9 ± 241.9				0.058
AUC_all_ (ng·h/mL)	25,697.2 ± 6510.9	30,333.7 ± 5401.5	31,302.6 ± 6186.3				0.121
AUC_inf_ (ng·h/mL)	26,261.5 ± 6660.0	30,940.4 ± 5461.7	31,922.5 ± 6233.0				0.124
Half-life (h)	12.9 ± 1.5	12.7 ± 1.2	12.5 ± 1.4				0.828
CL/F (L/h)	16.0 ± 3.3	13.3 ± 2.4	12.9 ± 2.2				0.049*
*ABCG2* c.34G > A	c.34 GG (n = 19)	c.34 GA (n = 6)	c.34AA (n = 2)				*p*-value
T_max_ (h)	4.0 ± 1.4	2.8 ± 0.7	3.0 ± 0.6				0.095
C_max_ (ng/ml)	1810.6 ± 720.37	1875.8 ± 445.11	1756.4 ± 319.86				0.791
AUC_all_ (ng·h/mL)	29,787.8 ± 15,368.2	28,562.9 ± 6403.8	28,328.0 ± 5857.5				0.957
AUC_inf_ (ng·h/mL)	30,402.8 ± 15,806.8	29,034.2 ± 6480.0	28,953.9 ± 5926.1				0.959
Half-life (h)	12.5 ± 1.0	12.1 ± 1.0	12.9 ± 1.4				0.436
CL/F (L/h)	15.2 ± 7.9	14.2 ± 2.8	14.3 ± 2.8				0.933
*ABCB1* c.1236C > T	*c.1236CC* (n = 6)	*c.1236CT* (n = 8)	*c.1236 TT* (n = 13)				*p-value*
T_max_ (h)	3.25 ± 0.52	3.25 ± 0.80	2.80 ± 0.63				0.246
C_max_ (ng/ml)	1958.5 ± 493.82	1724.8 ± 374.10	1746.1 ± 287.10				0.435
AUC_all_ (ng·h/mL)	31,530.6 ± 8564.2	28,312.5 ± 7579.7	27,192.4 ± 4244.7				0.403
AUC_inf_ (ng·h/mL)	32,079.9 ± 8634.5	28,952.8 ± 7807.0	27,771.9 ± 4281.3				0.420
Half-life (h)	12.1 ± 0.9	12.8 ± 1.4	12.9 ± 1.5				0.470
CL/F (L/h)	13.3 ± 3.7	14.7 ± 3.9	14.7 ± 2.3				0.615
*ABCB1* c.3435C > T	*c.3435CC* (n = 8)	*c.3435CT* (n = 14)	*c.3435 TT* (n = 5)				*p*-value
T_max_ (h)	3.12 ± 0.51	3.17 ± 0.66	2.5 ± 0.79				0.144
C_max_ (ng/ml)	1809.7 ± 466.98	1818.8 ± 351.34	1661.6 ± 217.44				0.708
AUC_all_ (ng·h/mL)	30,058.9 ± 7842.8	28,572.1 ± 6455.8	25,740.9 ± 2861.5				0.513
AUC_inf_ (ng·h/mL)	30,734.4 ± 7932.7	29,162.9 ± 6534.7	26,196.1 ± 3022.5				0.488
Half-life (h)	12.8 ± 1.49	12.8 ± 1.36	12.0 ± 1.00				0.512
CL/F (L/h)	13.8 ± 3.4	14.4 ± 3.3	15.4 ± 1.7				0.658
*ABCB1* c.2677G>T/A	*c.2677 GG* (n = 3)	*c.2677 GT* (n = 11)	*c.2677 GA* (n = 4)	*c.2677 TT* (n = 6)	*c.2677 TA* (n = 2)	*c.2677AA* (n = 1)	*p*-value
T_max_ (h)	3 ± 0.5	3.27 ± 0.71	3.12 ± 0.62	2.58 ± 0.73	2.75 ± 0.35	3.5	0.454
C_max_ (ng/ml)	1821.3 ± 517.3	1824.4 ± 367.51	1905.6 ± 509.56	1752.7 ± 295.99	1593.1 ± 16.899	1391.33	0.826
AUC_all_ (ng·h/mL)	31,980.4 ± 10,231.3	28,958.3 ± 6673.3	30,676.8 ± 6850.4	27,233.8 ± 4463.5	23,384.4 ± 1866.6	21,823.3	0.584
AUCinf (ng·h/mL)	32,675.0 ± 10,302.0	29,596.6 ± 6743.3	31,428.0 ± 6858.7	27,697.2 ± 4563.7	23,757.3 ± 1849.6	22,138.6	0.545
Half-life (h)	12.8 ± 2.0	13.1 ± 1.4	13.2 ± 1.3	12.0 ± 0.9	11.8 ± 0.4	11.5	0.423
CL/F (L/h)	13.0 ± 4.11	14.2 ± 3.5	13.2 ± 3.0	14.7 ± 2.3	16.8 ± 1.3	18.1	0.432

C_max_, maximum concentration; T_max_, time required to reach the maximum concentration; AUC_all_, total areas under the plasma concentration–time curve, AUC_inf_, areas under the plasma concentration–time curve with extrapolation to infinity; K_e_, elimination constant, CL/F, oral clearance, Vd/F, volume of distribution.

*p< 0.05 by ANOVA (Kruskal-Wallis) with posthoc (Tukey’s) comparisons.

### Effects of AGP Levels on Imatinib Pharmacokinetics

**FIGURE 1:**
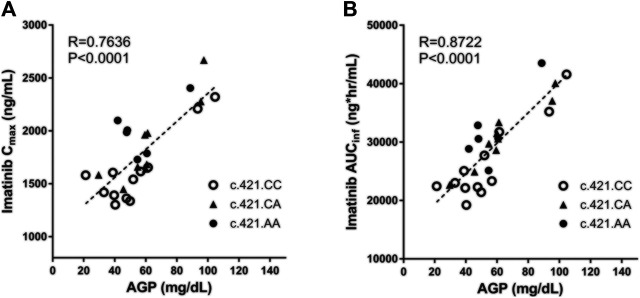
The correlation between individuals’ AGP level and their pharmacokinetic parameters (C_max_ and AUC_inf_) with respect to *ABCG2* c.421C>A genotype.

Plasma AGP levels exhibited a strong correlation with the pharmacokinetic parameters of imatinib. In particular, plasma AGP levels exhibited a significant positive correlation with C_max_ (Pearson *r* = 0.764, *p* < 0.0001) and AUC_inf_ (Pearson *r* = 0.872, *p* < 0.0001) and a significant and negative correlation with CL/F (Pearson *r* = −0.667, *p* < 0.001) (Figure 1; Table 3). No association was found with T_max_ or half-life.

**TABLE 3:** Correlation between individual alpha-1-acid glycoprotein (AGP) level and the respective pharmacokinetic parameters of imatinib.

	Pearson *r* correlation coefficient	*p*-value
T_max_	0.3753	0.053
C_max_	0.7636	**<0.0001**
AUC_all_	0.8681	**<0.0001**
AUC_inf_	0.8722	**<0.0001**
Half-life	0.0644	0.750
CL/F	−0.6673	**<0.001**

*p< 0.05

### Effects of Polymorphic ABCB1 and ABCG2 Genotypes Based on the Stratified AGP Levels

**FIGURE 2:**
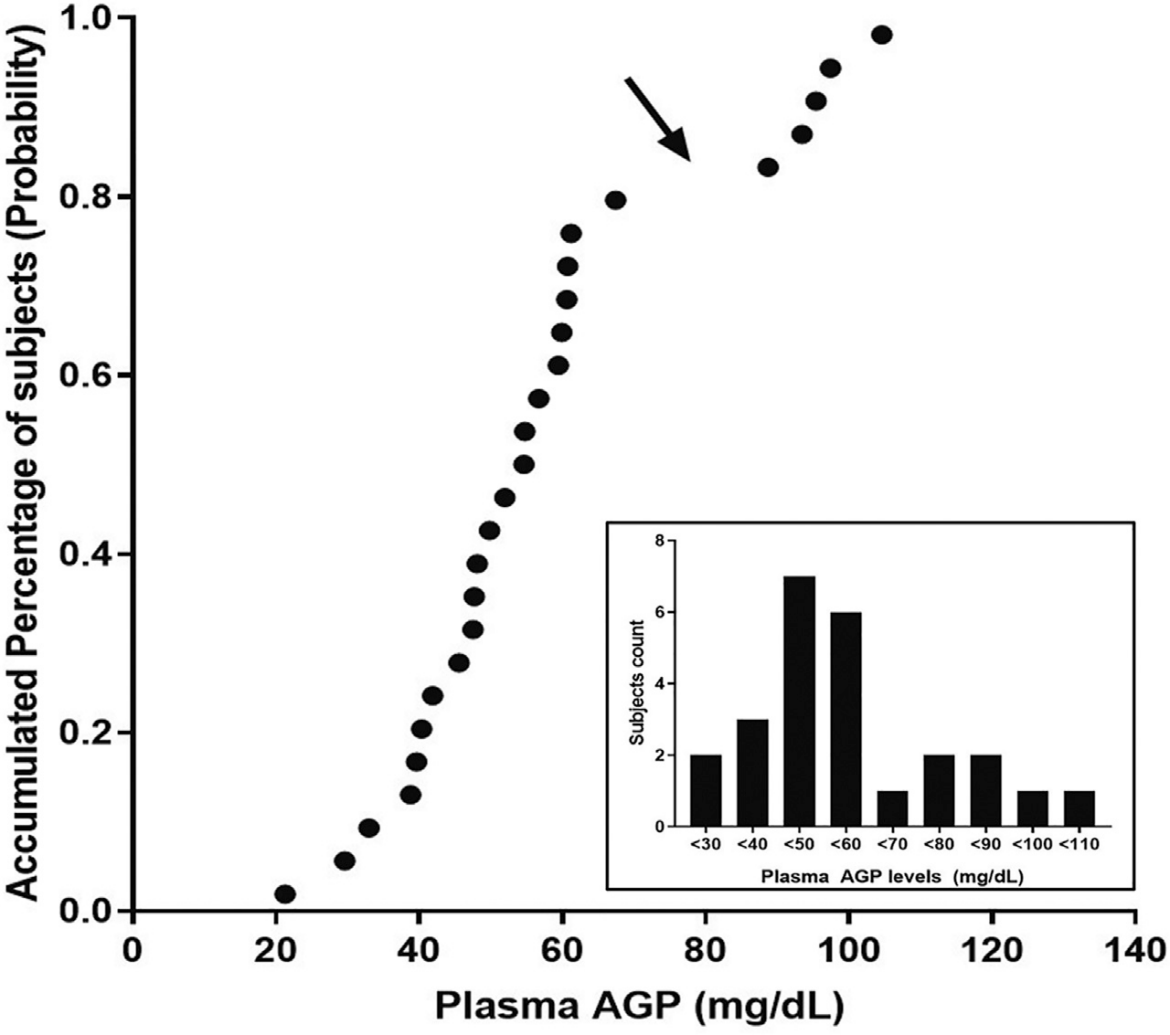
Probability plot for the plasma AGP levels observed in this study. The arrow points at the antimode, which determines normal and higher AGP levels in this population. The inset shows the distribution of the observed AGP levels in the population.

**FIGURE 3:**
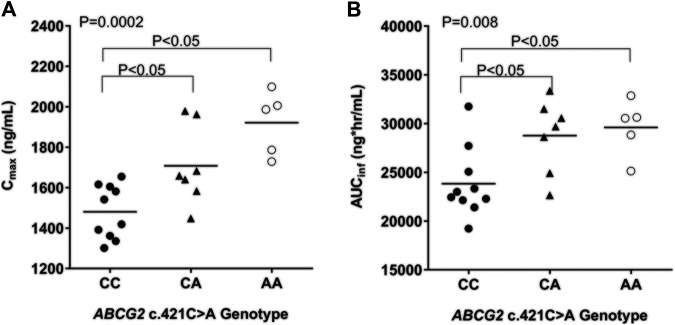
Comparison of pharmacokinetic parameters [C_max_ (A) and AUC_inf_ (B)] by *ABCG2* c.421C>A genotype in subjects with plasma AGP less than 80 mg/dl. ANOVA (Kruskal-Wallis test) with posthoc comparisons (Tukey's) were performed.

An evaluation of the plasma AGP distribution revealed a bimodal distribution with an antimode at 80 mg/dl (Figure 2). We, therefore, re-evaluated the imatinib pharmacokinetics with respect to the *ABCB1* and *ABCG2* polymorphisms after the exclusion of five subjects with AGP values above the antimode (≥80 mg/dl, n = 5). After this adjustment, *ABCG2* c.421C>A substantially influenced imatinib pharmacokinetics in this population: the *ABCG2* c.421C>A genotype increased the imatinib C_max_ values to 14,801.7 ng/ml for c.421CC, 1708.1 ng/ml for c.421CA, and 1921.4 ng/ml for c.421AA (*p* < 0.001). In addition, the average AUC_inf_ value was 23,835.6 ng h/ml for c.421CC, 28,764.3 ng h/ml for c.421CA, and 29,605.3 ng h/ml for c.421AA (*p* = 0.008) and their average CL/F values were 17.1 L/h, 14.1 L/h, and 13.6 L/h, respectively (*p* = 0.007), while no association was found in T_max_, and CL/F (Table 4; Figure 3, Supplementary Figure S1). We could not find any meaningful results in *ABCG2* c.34G>A.

**TABLE 4:** Comparison of pharmacokinetic parameters of imatinib by *ABCG2* genotypes in lower AGP levels (<80 mg/dl, n = 22).

*ABCG2* c.421C > A	c.421CC (n = 10)	c.412CA (n = 7)	c.421AA (n = 5)	Difference between genotypes (mean difference (90% CI of difference))			*p*-value
				CC *vs*. CA	CC *vs*. AA	CA *vs*. CC	
T_max_ (h)	2.9 ± 0.4	3.0 ± 0.9	2.9 ± 0.7	−0.1 (−0.8 – 0.6)	0.0 (−0.8 – 0.8)	0.1 (−0.7 – 0.9)	0.942
C_max_ (ng/ml)	14,801.7 ± 132.1	1708.1 ± 195.4	1921.4 ± 156.5	−227.0 (−399.1 – −55.6)	−440.7 (−631.5 – −249.8)	−213.3 (−417.3 – −9.3)	**<0.001** ^a**,**b**,**c^
AUC_all_ (ng·h/mL)	23,301.4 ± 3401.9	28,141.3 ± 3592.7	28,985.9 ± 2753.8	−4840.3 (−8433.1 – −1247.2)	−5685 (−9678.4 – −1691.1)	−844.6 (−5114.3 – 3424.2)	**0.006** ^a,b^
AUC_inf_ (ng·h/mL)	23,835.6 ± 3566.2	28,764.3 ± 3761.4	29,605.3 ± 2879.5	−4929.6 (−8692.4−1165.1)	−5770.2 (−9953.6 – −1587.0)	−841.0 (−5313.3 – 3630.9)	**0.008** ^a**,**b^
Half-life (h)	13.0 ± 1.5	12.9 ± 1.3	12.7 ± 1.4	0.0 (−1.5 – 1.6)	0.3 (−1.5 – 2.0)	0.2 (−1.7 – 2.1)	0.949
CL/F (L/h)	17.1 ± 2.3	14.1 ± 2.0	13.6 ± 1.4	3.0 (0.8 – 5.2)	3.5 (1.0 – 5.9)	0.5 (−2.1 – 3.1)	**0.007** ^a,b^
*ABCG2* c.34G>A	c.34 GG (n = 16)	c.34 GA (n = 5)	c.34AA (n = 1)	GG *vs.* GA	GG *vs.* AA	GA *vs.* AA	*p*-value
T_max_ (h)	3.0 ± 0.6	2.8 ± 0.7	3.0	0.2 (−0.5 – 0.9)	0.0 (−1.5 – 1.4)	−0.2 (−1.7 – 1.3)	0.878
C_max_ (ng/ml)	1655.2 ± 236.3	1717.2 ± 242.8	1301.2	−62.0 (−320.9 – 196.8)	353.9 (−166.8 – 874.7)	416.0 (−137.5 – 969.4)	0.303
AUC_all_ (ng·h/mL)	26,236.4 ± 4031.4	26,372.2 ± 3907.4	18,920.8	137.1 (−5090.2 – 5365.3)	7589.6 (−2929.1 – 18,106.0)	7451.4 (−3726.0 – 18,628.6)	0.228
AUC_inf_ (ng·h/mL)	26,841.9 ± 4150.1	26,826.9 ± 3993.2	19,225.6	321.3 (−4322.4 – 4964.7)	7923.0 (−1419.9 – 17,264.0)	7601.1 (−2326.6 – 17,528.4)	0.224
Half-life (h)	13.0 ± 1.4	12.1 ± 1.0	11.8	1.0 (−0.8 – 2.8)	1.4 (−2.2 – 4.9)	0.4 (−3.4 – 4.2)	0.355
CL/F (L/h)	15.1 ± 2.4	15.2 ± 2.1	20.8	−0.1 (−2.7 – 2.5)	−5.7 (−11.0 – 0.5)	−5.6 (−11.2 – −0.1)	0.091

C_max_, maximum concentration; T_max_, time to reach maximum concentration; AUC_all_, total area under the plasma concentration-time curve, AUC_inf_, area under the plasma concentration-time curve with extrapolation to infinity; K_e_, elimination constant; CL/F, oral clearance.

^a^ *p* < 0.05 by ANOVA (Kruskal-Wallis) with posthoc (Tukey’s) comparisons between c.421CC and c.421AA.

^b^ *p* < 0.05 by ANOVA (Kruskal-Wallis) with posthoc (Tukey’s) comparisons between c.421CC and c.421CA.

^c^ *p* < 0.05 by ANOVA (Kruskal-Wallis) with posthoc (Tukey’s) comparisons c.421CA and c.421AA.

## Discussion

The results of the present study indicated that *ABCG2* c.421C>A single nucleotide polymorphism significantly influenced imatinib pharmacokinetics in relatively low AGP plasma levels. However, neither the *ABCG2* nor the *ABCB1* polymorphism independently affected imatinib pharmacokinetics suggesting that the plasma AGP level was an important factor in the interindividual variability of imatinib pharmacokinetics.

The results of the present study showed a strong correlation between plasma AGP levels, and imatinib exposure and disposition, this was in agreement with previous literature (Widmer et al., 2006; Petain et al., 2008; Haouala et al., 2013), suggesting that the plasma AGP level was an important factor in the imatinib pharmacokinetics (Huang and Ung, 2013).

Based on our present study results, *ABCG2* c.34G>A polymorphism did not affect imatinib pharmacokinetics, whereas the *ABCG2* c.421C>A genotype significantly influenced the imatinib CL/F (*p* = 0.049), and showed a gene-dose dependent effect on the imatinib C_max_ and AUC values in the low AGP plasma levels group. Moreover, the participants who had more than one SNPs causing loss of function of ABCG2 and/or ABCB1 (not including *ABCG2* c.421C>A) did not show any tendency to affect imatinib pharmacokinetics, while the participants who only had *ABCG2* c.421C>A mutation with no other additional SNPs were shown to have a more discriminative effect (data not shown). These results suggest that *ABCG2* c.421C>A is a moderate factor in the modulation of imatinib pharmacokinetics. Although previous studies showed similar results regarding the effect of *ABCG2* c.421C>A on the imatinib pharmacokinetics (Breedveld et al., 2005), some studies showed a significant difference in imatinib pharmacokinetics only in the dominant model (i.e., c.421 CC + CA vs. AA) or a gene-dose dependent effect on imatinib pharmacokinetics with no statistical significance (Petain et al., 2008; Takahashi et al., 2010; Seong et al., 2013). Given the discrepancies between the results of previous studies and those of the present study, we can postulate, *a priori*, that other factors may mask the effect of the ABCG2, including plasma AGP levels and demographic characteristics (Takahashi and Miura, 2011). However, in this study, the effects of age, body weight, and BMI on imatinib pharmacokinetics were unlikely to play a substantial role. Similarly, previous studies did not show any relationship with demography (Larson et al., 2008; Takahashi and Miura, 2011). When we analyzed plasma AGP distribution, the probability plot showed a bimodal distribution. Considering the strong positive correlation between imatinib pharmacokinetics and plasma AGP levels, we hypothesized that higher plasma AGP levels concealed the genetic effects on imatinib pharmacokinetics. Interestingly, *ABCG2* c.421C>A genotypes substantially influenced imatinib pharmacokinetics in subjects with plasma AGP levels below 80 mg/dl, while other genotypes did not. This phenomenon suggests that plasma AGP levels and ABCG2 simultaneously influence imatinib pharmacokinetics, but that the role of ABCG2 might be masked in subjects with higher plasma AGP levels. We supposed that a low concentration of AGP contributed to a relatively increased fraction of free plasma imatinib levels, and thereby more easily affected by the functional *ABCG2* polymorphism (Bohnert and Gan, 2013).

It has been suggested that imatinib is a substrate of P-glycoprotein expressed by *ABCB1* (Gurney et al., 2007; Vivona et al., 2012). *ABCB1* genetic polymorphisms, including c.1236C>T, c.2677G>T/A, and c.3435C>T, influence its disposition and clinical response to various *ABCB1* substrates; in addition, it has been suggested that these polymorphisms modulate the plasma levels of imatinib as a substrate of P-glycoprotein (Burger and Nooter, 2004; Gurney et al., 2007; Vivona et al., 2012). However, a previous meta-analysis showed that neither *ABCB1* c.2677G>T(A) nor c.3435C>T was a risk factor for poor clinical response of imatinib treatment in Asian CML patients, whereas c.1236C>T was a risk factor in Asian but not Caucasian CML patients (Zu et al., 2014; Zheng et al., 2015). In the present study evaluation of the effects of polymorphisms on imatinib pharmacokinetics showed no statistically significant effects on imatinib disposition. Similarly, other studies concluded that none of these polymorphisms influenced imatinib pharmacokinetics (Petain et al., 2008; Seong et al., 2013); Dickens et al. revealed that the SNPs did not affect the transporter activity of human P-glycoprotein *in vitro* (Dickens et al., 2013). We, therefore, suggested that *ABCB1* polymorphisms might play a minor role in the disposition of imatinib.

Our study has some limitations. First, we assessed the pharmacokinetics of imatinib in healthy subjects with various *ABCG2* and *ABCB1* genotypes after a single dose of imatinib, even though imatinib is a long-term anticancer drug. However, to assess the pharmacogenetic effects only, we recruited a relatively homogenous group without other confounding factors that could affect imatinib pharmacokinetics (Takahashi and Miura, 2011). Secondly, while it has been suggested that various cytochrome P450 isoforms (CYP3A4/5, CYP1A2, CYP2C9, and CYP2C19) are involved in imatinib disposition (Weiner et al., 2007), in this study, we only assessed the pharmacogenetic effects of *ABCG2* and *ABCB1* polymorphisms. Although we did not assess the effect of polymorphisms of these genes, recently published findings have demonstrated their minor contributions (Gardner et al., 2006; Gurney et al., 2007). Another limitation of this study is the low sample size, which was inevitable to perform a thorough pharmacokinetic study design.

In conclusion, we observed that plasma AGP levels were strongly correlated with imatinib pharmacokinetics, and *ABCB1* polymorphisms did not influence imatinib pharmacokinetics. However, the *ABCG2* c.421C>A polymorphism plays a substantial role in imatinib pharmacokinetics in subjects with low plasma AGP levels; this implied that its effect on the imatinib pharmacogenetics was masked at higher AGP plasma levels.

## Data Availability

The data analyzed in this study is subject to the following licenses/restrictions: The datasets presented in this article are not readily available to protect participants’ privacy. Requests to access the datasets should be directed to JP. Requests to access these datasets should be directed to JP, jypark21@korea.ac.kr.
