# Supplementary figures and images for: ABCG2 Single Nucleotide Polymorphism Affects Imatinib Pharmacokinetics in Lower Alpha-1-Acid Glycoprotein Levels in Humans
Source: Front Pharmacol. 2021 Apr 29;12:658039. doi: 10.3389/fphar.2021.658039 (PMC8116740; doi:10.3389/fphar.2021.658039)

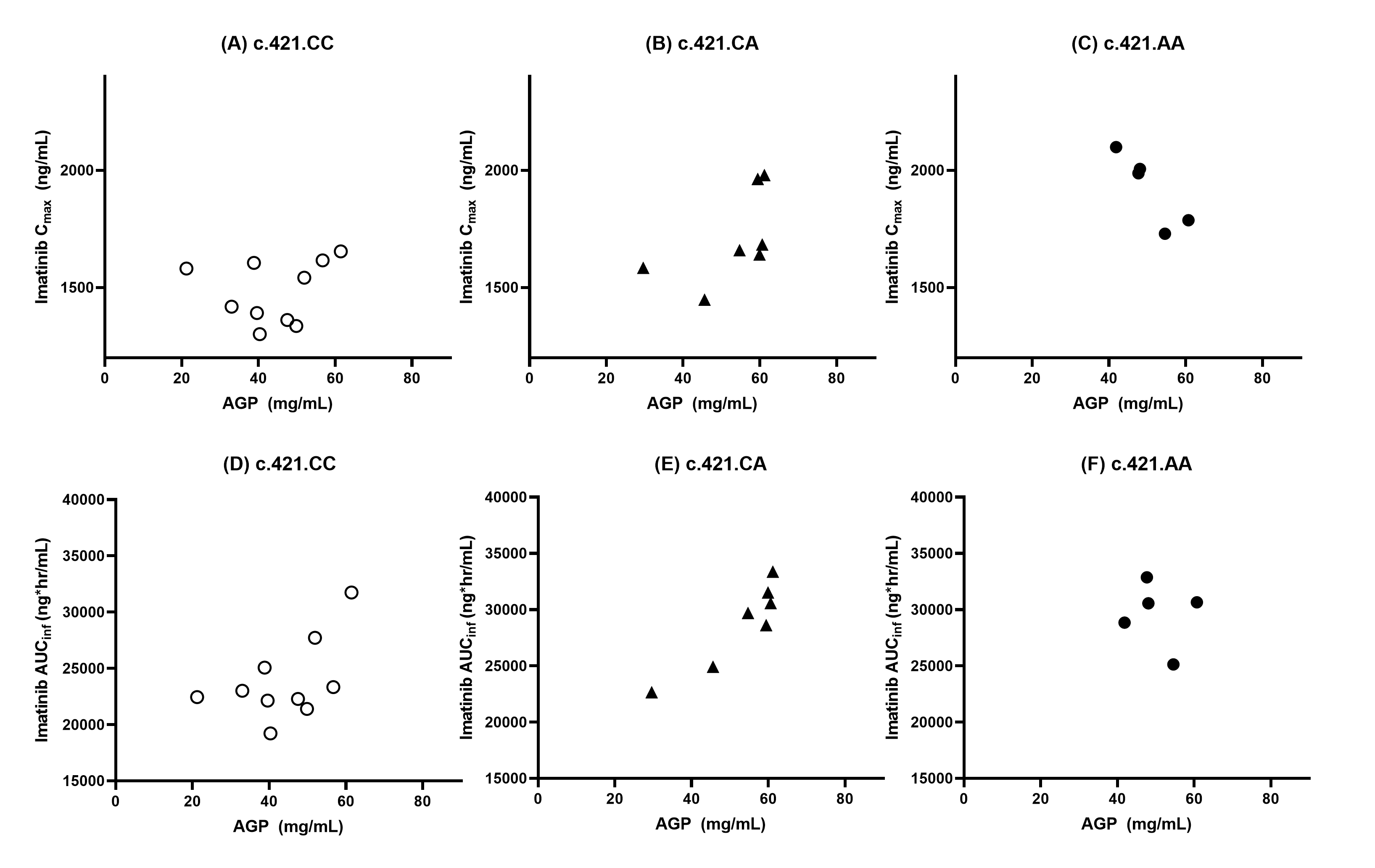

Supplement: Supplementary file 1 [file Image1.TIF]
